# The global cancer genomics consortium's third annual symposium: from oncogenomics to cancer care

**Published:** 2014-03

**Authors:** Luis Costa, Sandra Casimiro, Sudeep Gupta, Stefan Knapp, M.Radhakrishna Pillai, Masakazu Toi, Rajendra Badwe, Maria Carmo-Fonseca, Rakesh Kumar

**Affiliations:** ^1^ Instituto de Medicina Molecular, Faculdade de Medicina, Universidade de Lisboa, Lisbon, Portugal; ^2^ Hospital de Santa Maria – CHLN, Lisbon, Portugal; ^3^ Tata Memorial Centre, Mumbai, India; ^4^ Structural Genomic Consortium, University of Oxford, Oxford, UK; ^5^ Rajiv Gandhi Center for Biotechnology, Kerala, India; ^6^ Kyoto University Graduate School of Medicine, Japan; ^7^ The George Washington University, Washington, DC, USA; ^*^ Member, The Global Cancer Genomics Consortium, Department of Biochemistry and Molecular Medicine, the George Washington University, Washington, DC, USA

**Keywords:** Oncogenomics, Cancer Biomarkers, Cancer Therapy

## Abstract

The Global Cancer Genomics Consortium (GCGC) is a cohesive network of oncologists, cancer biologists and structural and genomic experts residing in six institutions from Portugal, United Kingdom, Japan, India, and United States. The team is using its combined resources and infrastructures to address carefully selected, shared, burning questions in cancer medicine. The Third Annual Symposium was organized by the Institute of Molecular Medicine, Lisbon Medical School, Lisbon, Portugal, from September 18 to 20, 2013. To highlight the benefits and limitations of recent advances in cancer genomics, the meeting focused on how to better translate our gains in oncogenomics to cancer patients while engaging our younger colleagues in cancer medicine at-large. Over two hundreds participants actively discussed some of the most recent advances in the areas cancer genomics, transcriptomics and cancer system biology and how to best apply such knowledge to cancer therapeutics, biomarkers discovery and drug development, and an essential role played by bio-banking throughout the process. In brief, the GCGC symposium provided a platform for students and translational cancer researchers to share their excitement and worries as we are beginning to translate the gains in oncogenomics to a better cancer patient treatment.

## INTRODUCTION

The Global Cancer Genomics Consortium (GCGC) functions as a cohesive interface that aligns a multidisciplinary team of cancer and structural biologists, computational genomics experts, and oncologists to address shared global cancer research challenges through the application of high-throughput technologies using resources from its network. The GCGC is a dynamic initiative connecting members and their institutions from Portugal, United Kingdom, Japan, India and United States. The GCGC network holds an annual meeting to discuss and debate the most pressing issues in cancer research and treatment, share their recent findings, exchange research ideas, and train students, young scientist and faculty members, in cutting-edge cancer genomics and need of cancer patients. The Third Annual Symposium was held at the Institute of Molecular Medicine, Lisbon Medical School, Lisbon, Portugal, from September 18 to 20, 2013, attracted 215 participants, and counted with 25 invited platform speakers and 29 poster presenters. Once again, the GCGC Symposium brought together a highly motivated group of clinical and research cancer specialists and scientists, students and young researchers to share their latest results and ideas for translating the benefits of post-genomic advances in cancer medicine to improve the life of cancer patients.

### Focus on the Clinical Relevance of Oncogenomics

The third meeting was focused on the integration of the knowledge and translational strategies in the post–genomic era toward its clinical applicability. The symposium was opened by Prof. Luis Costa from the Institute of Molecular Medicine and Hospital de Santa Maria, and the introductory welcome remarks by Prof. Lobo Antunes from the same institution. Profs. Costa and Lobo Antunes shared the message of the Annual GCGC Symposium, emphasizing the importance of assessing the clinical relevance of knowledge that is being generated in post-genome-sequencing era with an overall goal to develop effective diagnostic and therapeutic outcomes for cancer patients. The meeting was organized into eight platform sessions and one poster session, and included two keynote lectures. The symposium focused on eight major scientific and clinical themes: 1) breast cancer genomics and transcriptomics: where do we stand?; 2) current application of genomics in cancer therapy; 3) genomic approaches to facilitate biomarkers discovery and drug development; 4) genomics in clinics: biobanking; 5) innovative clinical trial designs in the era of cancer genomics; 6) application of cancer genomics research; 7) cancer system biology: cancer transcriptome, proteome and metabolome; and 8) how to integrate microenvironment and metabolomics with cancer genomics. The meeting concluded with a panel discussion about the future and next steps of GCGC research projects.

### Breast Cancer Genomics and Transcriptomics: Where do We Stand?

The theme was introduced by the opening keynote lecture by Dr. Clifford Hudis from the Memorial Sloan-Kettering Cancer Center, and President of American Society of Clinical Oncology (ASCO). Dr. Hudis focused on the current role and future prospects of genomics and highlighted the need to broaden our understanding of genomic changes including complex and interacting alterations, functional alterations without conventional structural changes in solid tumors. He also stressed the possibility of considering even alternative or complimentary explanations for the growth of some malignancies. Dr. Hudis reinforced that clinicians and researchers need to be inclusive of a broader patient base and tumor acquisition supported by an improved bioinformatics for its eventual utility by all. Dr. Hudis concluded his lecture by sharing the ASCO's CANCER LINQ project.

Dr. Eric Winer from the Dana-Farber Cancer Institute focused on the contribution of genomics to the molecular diagnosis of breast cancer patients and their treatments. Until this point, the convergence of clinical and genomic data led to four families of breast cancer: the “basal-like”, the HER2; the Luminal B (estrogen positive with high proliferative index) and the Luminal A (estrogen positive with low proliferative index). Up to this moment, this sub-classification can be used to assess prognosis and to determine appropriate treatment toward a more personalized therapy. It has been also useful to drive research in order to assess changes in treatment response to each one of the sub-types, as well as to characterize better mechanisms of resistance to the available treatments and discover new “druggable” targets. Single gene changes responsible for somatic alterations or germ line mutations are also under the most desirable new findings in genomic research. For example, HER-2 and BRCA genes alterations are the most relevant gene alterations with clinical impact. Mutations in the PI3K pathway are among the most common mutations in breast cancer, particularly in ER positive and in HER2 positive tumors. However, despite of the fact that a large number of new drugs are in pipeline, only a handful options are advancing to the clinical scenario.

Dr. Rakesh Kumar from the George Washington University focused on how RNA sequencing efforts could provide new insights into breast cancer transcriptome. The McCormick Genomic and Proteomic Center (MGPC) team has recently completed an extensive comparative analyses of triple-negative breast, estrogen receptor positive, and HER2 positive breast cancers and presented a comprehensive digital transcriptome [1]. This work led to identification of novel and unannotated transcripts, breast cancer sub-type specific transcriptomic adaptations, and clues about new set of modulators of breast cancer. Using in-house pipelines, the team also delineated splicing signatures and differentially spliced genes in human transcriptome. In general, distinct patterns of primary transcripts and promoter switching in breast cancer were identified and these molecular changes might contribute to the noted heterogeneity of breast cancer transcriptome. Using the same RNA-sequencing primary datasets, algorithms were also developed to recognize the genetic variance of breast cancer in the context of its allelic preferential expression, and splicing signatures. Together with Drs. Badwe and Gupta from the Tata Memorial Center Mumbai, Prof. Kumar's team unlocked the transcriptomic insights of breast tumors from a subset of breast cancer patients were treated with a single injection of progesterone prior to the surgery [2]. Dr. Kumar also presented results from on-going collaboration with the Institute of Molecular Medicine in Lisbon, where the team is revealing the transcriptome of bilateral breast cancer with different tumor types.

Dr. Caterina Marchió from the Turim University addressed the gap between genomics and molecular pathology in the management of patients with breast cancer. Dr. Marchió highlighted new promises and challenges involved in on-going rapid integration of massive parallel next generation sequencing into clinical arena. In order to fill in the gap between genomics and modern pathology (which appears still conspicuous), tests will definitely play a role in diagnostic breast cancer pathology, it will be important for pathologists to be ready to integrate such developments into diagnostic breast cancer pathology while ensuring the best clinical management of breast cancer patients.

To conclude this session, Dr. Nikhil Wagle from the Dana-Farber Cancer Institute discussed the use of systematic genomic profiling approaches – including hotspot genotyping, targeted massively parallel sequencing, and whole exome sequencing – to better understand the molecular determinants of tumorigenesis, characterize mechanisms of therapeutic response and resistance, and identify actionable genomic alterations to aid with clinical decision-making. These approaches include hotspot genotyping, targeted massively parallel sequencing and whole exome sequencing. Dr. Wagle also highlighted the need to test the “genomics-driven” cancer medicine hypothesis through novel clinical trial design and centralized shared databases [3,4]. Dr. Wagle presented the initial results from the CanSeq initiative, collaboration between the Broad Institute, Dana-Farber, and Brigham and Women's Hospital, on the prospective whole-exome sequencing for patients with for the benefits of clinically actionable results [5]. To conclude Dr. Wagle reinforced the notion that genomic profiling technologies are changing the way we will practice oncology by identifying new therapeutic opportunities as we can now identify common and rare genomic alterations (“the long tail”) in patients with cancer that might predict responses to novel targeted therapies. Although efforts are underway to implement tumor genomic profiling platforms in the clinical arena, the interpretation of genomic alterations and the incorporation of such data into clinical care continue to be challenging. The “genomics-driven” cancer medicine hypothesis is beginning to be tested – we need clinical trials and shared databases to best answer the question.

### Application of Genomics in Cancer Therapy

The meeting brought together three leading experts to discuss emerging applications of genomic-driven findings to the cancer therapy. Dr. Carmo-Fonseca attempted to link cancer with altered expression of proteins implicated in the regulation of splicing machinery and suggested such molecules as potential targets for anticancer treatment. Dr. Carmo-Fonseca also shared some innovative data about the identification of splicing factors that are mis-regulated in cancers. Recurrent mutations in genes encoding essential components of the splicing machinery such as *U2AF1* and *SF3B1* are related to cancer. Dr. Carmo-Fonseca focused on the potential role of spliceostatin in blocking the formation of a catalytic spliceosome and raised the possibility of using such approaches in cancer therapeutics [6].

This was followed by Dr. Sudeep Gupta from the Tata Memorial Hospital presented a genetic/genomic perspective on gynecological cancers. Dr. Gupta focused on focus on ovarian cancer with a particular emphasis on high grade serous subtype. Dr. Gupta presented recent studies dissecting the molecular heterogeneity of ovarian cancer [7], and contributing to the new classification of ovarian cancer into the so called Type 1 and Type 2 tumors [8]. From the genomic perspective, he highlighted the TCGA initiative and outlined the progress in our understanding of differences between different types of ovarian cancer in clinical presentation, genetic predisposition, hallmark molecular abnormalities, response to treatment and outcome.

Dr. Raquel Seruca from IPATIMUP (Institute of Molecular Pathology and Immunology at the University of Porto) review the severe effects caused by *CDH1* pathogenic variants and discuss how these novel findings can be applied for the development of novel screening tools and the development of therapeutic strategies to treat patients harbouring carcinomas associated with the loss of E-cadherin. *CDH1* germ line mutations and somatic alterations cause hereditary diffuse gastric cancer and have been extensively studied by Dr. Seruca and colleagues [9].

### Genomic Approaches to Facilitate Biomarkers Discovery and Drug Development

The relevance of genomics in biomarkers and drug development was addressed in the next presentations. Dr. Masakazu Toi from the Kyoto University School of Medicine focused on how the development of new predictive biomarkers could help to maximize the treatment efficacy and mimic the burdens toxicity and cost of anti-HER2 therapy in breast cancer. The list of validated markers to predict treatment response continues to be limited considering the wealth of genomic information available. The search for markers which could effectively predict of response to MTOR inhibitors is on. This area of development is focused on the role of S6 kinase, PTEN status and PI3K mutation to predict the response to mTOR inhibitors. Dr. Toi underlined the importance of inter-observer concordance for IHC tests, such as the Ki-67 proliferative index in breast cancer as the reliability of Ki67 test in grade 2 tumors is not very clear at the moment as compared to high-expressing grade 3 tumors.

Dr. João Nuno Moreira from the Center for Neuroscience and Cell Biology of University of Coimbra presented a new rationale for the to development of therapies -targeting tumor microenvironment on the basis of characterized mechanisms while improving access to intracellular sites of actions. Dr. Moreira stressed the potential of ligand-mediated targeted delivery as well as nanotechnologies-based approaches in the treatment of solid tumors such as breast cancer [10]. The systemic delivery of siRNA was also addressed [11].

Dr. Radhakrishnan Pillai from the Rajiv Gandhi Center of Biotechnology addressed the significance of cancer stem cell hypothesis, which puts forth that cancer cells have a hierarchical developmental structure in which only a fraction of cells termed cancer stem cells (CSCs) can proliferate indefinitely to form tumors. Dr. Pillai presented preliminary evidences for rare escape of tumor cells from drug induced cell death, after an intermediate stay in a non-cycling senescent stage followed by unstable multiplication. Dr. Pillai's data suggested indicated that rare cancer cells escape from drug induced caspase activation by entering into a high ROS quiescence state followed by re-activation of anti-oxidant systems to help them to stabilize several transcription factors (such as NRF2 & OCT 4) and stem cell markers. Long term chemical hypoxia treatment could lead to expansion of drug efflux population and stabilization of HIF1-alpha while cells escaping hypoxia tend to be more invasive. Dr. Pillai concluded by presenting some new data on the use of this knowledge to design better screening methods for anti-cancer drug discovery.

This session was ended by Dr. Luís Costa's presentation, emphasizing the need of biology-driven clinical research to drug development wherein both the target and the disease model are equally important. Prof. Costa stressed that the success of biology-driven trials will be dependent on the discovery of reliable biomarkers. Dr. Costa believes that one of the great challenges to accelerate drug development in cancer care is the promotion of transversal-biology at the clinical and scientific level. Important information driven from biology and from clinical research observation can be transferred across different tumor types that share common driven-events at different stages. Tumor-host bio-banking designed to address these hypothesis is crucial to support a transversal-translational-research. Dr. Costa also pointed how the GCGC mission to foster the creation of these multidisciplinary research teams an earlier intervention at the pre-graduation level would be desirable to develop both clinicians and scientists with transferable language and tasks.

### Genomics in Clinics -Bio-banking

The theme was introduced by the opening keynote lecture by Dr. Carlos Caldas from the Cancer Research UK. Dr. Caldas made an exciting presentation highlighting the molecular characterization of 2,000 breast cancers to illustrate stratification of breast cancer into 10 subtypes with distinct biology and clinical outcomes. Genome-wide copy number profiling of 997 tumors has revealed additional heterogeneity within the intrinsic sub-types of breast cancer. Joint clustering of copy number and expression data lead to a new molecular taxonomy of breast cancer. Such an approach improved prediction of outcome in ER+/HER2- cases and exhibited distinct prevalence and pattern of metastasis and sub-groups [12-14].

The remainder of the session focused on the strict connection between research-driven tumor bio-banking and the translational research outcomes. Dr. Fátima Carneiro from IPATIMUP and Hospital de São João emphasized a crucial role that pathologists in translational research by the establishment of a bridge between clinicians and basic researchers. Dr. Carneiro demonstrated why tumor banks are a vital resource for cancer research, using the example if the Portuguese National Network of Tumor Biobanks.

Dr. Sandra Casimiro from Instituto de Medicina Molecular focused on her experience in colorectal cancer, bone metastases and breast cancer bio-banking. Dr. Casimiro presented a workflow for a successful strategy in tumor bio-banking, and demonstrated the different potential of project-driven tumor bio-banking versus the broad prospective collection of tumor specimens by a dedicated structure like Biobank IMM [15].

Next, Dr. Cláudia Faria from the Labatt Brain Tumor Research Center and Hospital de Santa Maria explained bio-banking primary brain tumors and brain metastases. She also summarized the mission of the Medulloblastoma Advanced Genomics International Consortium (MAGIC) and collaborative bio-banking activities at IMM involving tumor tissue, blood and plasma samples as well primary stem cell culture.

### Innovative Clinical Trial Designs in the Era of Cancer Genomics

This session debated the impact of cancer genomics in a “new” design of clinical trials. Dr. Nancy Lin from Dana-Farber Cancer Institute pointed the challenge in the screening and recruitment of patients in this new era of molecular stratification and addressed the question whether we are prepared or not to test new discoveries in cancer genomics. Testing new discoveries can be challenging for several reasons: testing is complex, requires novel methods only available in academic or other specialized laboratories, asks for reproducibility, the object of testing is not routine; requires research consent and retrieval of tissue; potentially more tissue is required, there is higher likelihood of failure to obtain results; and the risk of “using up” tissue; frequently the turnaround takes time like weeks to months. Most important, there is only preclinical evidence and limited phase I data before initiation of proof-of-concept studies [16]. Finally, Dr. Lin suggested that some (if not most) of genomics data will be valuable in identifying resistance mechanisms and new targets that could be applied more broadly rather than in directing the care of individual patients.

Dr. Alberto Bardelli from the University of Turim discussed on the molecular alterations in *KRAS*, *NRAS*, *BRAF* and *MET* and their association with the onset of acquired resistance to anti-EGFR blockade in colon cancer. Dr. Bardelli presented an optimized diagnostic platform to identify resistance-associated genetic alterations in the blood of patients (liquid biopsy) months before radiographic documentation of disease progression, providing the rationale for delaying or reversing resistance to anti EGFR therapies in colon cancer and such a model could be used for designing molecularly driven- clinical trials [17].

Dr. Ian Krop from Dana-Farber Cancer Institute approached the question whether the actual design of clinical trials is prepared to test the new discoveries of cancer genomics. The development of validated markers is crucial in this setting. The design of phase II biomarker-driven trials was discussed and sample size considerations were presented in the enrichment design and randomized block design of clinical studies. Most studies performed the biomarker analysis using primary tissues rather than therapy refractory samples. Dr. Krop suggested that the study of circulating tumor cells and circulating DNA can also provide relevant information about the progression of disease. The question of therapeutic resistance continues to be one of the most understudied areas with only a handful of validated mechanisms. Therefore, there is a strong need to evaluate metastatic tumor samples in refractory patients

### Cancer System Biology: Cancer Transcriptome, Proteome and Metabolome

Dr. Anelia Horvath from George Washington University shared latest GCGC data on characterization of the genetic variance of 17 tumors representing the most common breast cancer receptor subtypes: triple-negative (TNBC), non-TBNC and HER2-positive breast cancer, using whole transcriptome sequencing [1]. This study illustrates the power of RNA-sequencing in revealing the variation landscape of breast transcriptome and exemplifies analytical strategies to explore regulatory interactions among cancer relevant molecules.

Next Dr. Stefan Knap from the University of Oxford talked about how structural information has facilitated chemical biology programs that aim to develop selective inhibitors that can be used as tool molecules to study the role of kinases in RNA splicing and structural comparison of the generated crystal structures revealed structural features that can be utilized for the development of highly specific inhibitors. Dr. Knapp research on the development of selective kinase inhibitors has focused on Cdc2-like kinases (CLKs), which are splicing regulators and found to be deregulated in many cancers. The effects of specific inhibition of CLK1 on TS splicing in endothelial cells, the effect of SRPK2 on VEGF splicing, and the effect of DIRK inhibition, were presented as relevant examples of kinase inhibitors potential.

On the last presentation of this session Dr. Sérgio Dias from Instituto de Medicina Molecular presented recent data showing the metabolic alterations in microenvironment gives instructive cues that regulate/modulate the metastatic potential of cancer cells, using models of hematological and solid cancers. Dr. Dias focused on how a high fat systemic environment affects cancer behavior. He shared recent data showing that mice on hypercholesterolemic diet present bigger breast tumors, with higher proliferation, migration, and mesenchymal traits, and lower adhesion, more prone to progress into the central nervous system [18]. Dr. Dias has previously shown high cholesterol perturbs the bone marrow microenvironment [19]. He also presented data showing increased leukemia burden and spread on high cholesterol mice, with higher trans-endothelial migration, probably due to CX3CR1 induction [20].

### Need to Integrate Microenvironment and Metabolomics with Cancer Genomics

In the final session of this meeting, speakers highlighted the importance of tissue microenvironment on the comprehension and analysis of cancer genomics data. Dr. Sandra Casimiro from Instituto de Medicina Molecular focused on the ‘vicious cycle’ of bone metastases and on the need to identify and dissect the mechanisms and events involved in bone colonization within different sub-types of breast cancer. This can lead to new prognostic and predictive markers, and to new potential therapeutic molecules specifically targeting the tumor compartment of bone metastases. Dr. Casimiro showed how pre-clinical research with cancer cell lines and animal models is being translated into clinical models, by sharing gene expression signatures of metastases [15], and showed evidence that targeting RANKL-RANK pathway may also affect the tumor compartment of bone metastases [21].

Dr. Fátima Baltazar from the University of Minho talked about the expression of monocarboxylate transporters (MCTs) and the MCT1/4 chaperone CD147, in human cancers [22]. MCT isoforms 1 and 4 mediate the plasma membrane efflux of lactate coupled with a proton, playing an important role in the maintenance of the metabolic phenotype of tumours. The pattern of MCT expression varies with cancer types and MCT overexpression has important associations with tumor aggressiveness. In general, MCT inhibition in glycolytic tumour cells leads to a decrease in lactate production, cell proliferation, migration and invasiveness [23]. Although MCT activity is essential for the maintenance of the metabolic phenotype of tumours, however, MCTs are differentially expressed among the solid tumours and future strategies of MCT inhibition in cancer treatment should take this fact into account.

Dr. Bruno Silva-Santos from Instituto de Medicina Molecular focused on research on gamma-delta T lymphocytes, which play key, non-redundant anti-tumor roles in animal models of tumor development. Dr. Silva Santos also spoke about stress-inducible determinants of anti-tumor gamma-delta T cell responses and its implications for manipulating lymphocyte lineage in cancer immunotherapy. His work has identified markers of susceptibility versus resistance to gamma-delta T cell-mediated cytotoxicity. Expression and functional studies also identified the chemokine CCL2 as a major determinant of gamma-delta T cell infiltration into solid tumors and counter-receptor CCR2 to infiltrate tumors in vivo, where they inhibited tumor cell growth [24].

Finally, Dr. João Barata from Instituto de Medicina Molecular shared new data on how IL-7 and IL-7R constitute an important oncogenic axis in T-cell leukemia, underpinning the relevance that both cell-autonomous and cell extrinsic cues can have in promoting cancer. Dr. Barata showed how IL-7 accelerates human T-ALL expansion in vivo [25]; IL-7R mutational activation promotes T-ALL [26]; forced wild type IL7R expression appears to promote T-cell oncogenesis.

## CONCLUSIONS AND ON-GOING GCGC TRANSLATIONAL ENDEAVORS

In brief, the meeting fulfilled one of the original mission of GCGC to bring its members and associated institutions together to review the progress made during the last year while bring leaders in the field and local students and fellows together to also share their recent findings with the GCGC members at-large. Collectively, sessions were designed to share the latest information in the context of pertinent bottlenecks in a given sub-themes and present potential solutions to move forward. This exercise could be best exemplified by highlighting the significance of quality onco-biobanking for asking genomic questions as well as validating the outcome before it could be implemented to the bedside. There was also a lot of excitement to witnessing the changing culture in organizing multidisciplinary research teams of oncologists, scientists, pathologists and surgeons while asking meaningful translational cancer medicine questions. The meeting participants also felt the need of start better integrating cancer transcriptome with proteome to promote targeted cancer therapeutics. Drs. Luis Costa and Rakesh Kumar closed the meeting by announcing that the next meeting will focus on Epigenome and Cancer Medicine and the 4^th^ GCGC meeting and will be held at the Kyoto University Graduate School of Medicine, Kyoto, Japan in 2014.
